# TIPE3 promotes drug resistance in colorectal cancer by enhancing autophagy via the USP19/Beclin1 pathway

**DOI:** 10.1038/s41420-025-02477-x

**Published:** 2025-04-25

**Authors:** Chun Chen, Longyang Jin, Hong Wan, Hu Liu, Shuping Zhang, Gang Shen, Jiao Gong, Yong Zhu

**Affiliations:** 1 Department of General Surgery, Shanghai General Hospital, Shanghai Jiao Tong University School of Medicine, 200080 Shanghai, China; 2 Department of Colorectal Surgery, The Sixth Affiliated Hospital of Sun Yat-sen University, 510655 Guangzhou, China; 3 Department of General Surgery, The First Affiliated Hospital of Anhui Medical University, 230022 Hefei, China; 4 Department of Emergency Surgery, First Affiliated Hospital of Anhui Medical University; Anhui Public Health Clinical Center, 230022 Hefei, P.R. China; 5 Department of Laboratory Medicine, Third Affiliated Hospital of Sun Yat-sen University, 510630 Guangzhou, China; 6 Department of Hepatobiliary Surgery, Innovative Institute of Tumor Immunity and Medicine (ITIM), Anhui Province Key Laboratory of Tumor Immune Microenvironment and Immunotherapy, Anhui Provincial Innovation Institute for Pharmaceutical Basic Research, 230022 Hefei, Anhui China

**Keywords:** Colorectal cancer, Cancer microenvironment

## Abstract

Drug resistance is a major obstacle to the effective treatment of colorectal cancer (CRC). However, the underlying mechanism remains unclear. In this study, we investigated the function and mechanism of TIPE3 in drug resistance of CRC. TIPE3 expression in clinical samples and CRC cell lines was detected using qPCR. CCK-8 and colony formation assays were employed to analyze the proliferation of CRC cells. The apoptosis of CRC cells was analyzed using flow cytometry, and autophagy of CRC cells was detected using western blotting, transmission electron microscopy, and immunofluorescence. Moreover, the relationship between USP19 and Beclin1 was detected using co-immunoprecipitation. CRC cells that had been transfected with OE-TIPE3 were co-cultured with macrophages (THP-1 cells induced by PMA), to create a model of TIPE3 overexpression in macrophage M2 polarization. Additionally, a nude mouse tumor model was established. After chemotherapy, tumor apoptosis was detected using the TUNEL assay, and autophagy levels were measured using immunofluorescence, immunohistochemistry, and western blotting. TIPE3 expression was increased in both CRC tumors and cell lines. TIPE3 overexpression substantially promoted drug resistance in CRC in vivo and in vitro. Furthermore, TIPE3 upregulated USP19 protein expression, which accelerated autophagy. In addition, co-immunoprecipitation showed an interaction between USP19 and Beclin1. TIPE3 increased drug resistance by enhancing CRC cell autophagy via the USP19/Beclin1 pathway and stimulating macrophage polarization towards the M2-type. Thus, TIPE3 could serve as a potential target for the development of novel strategies to overcome chemoresistance.

## Introduction

Colorectal cancer (CRC) is the third most commonly diagnosed form of cancer globally, accounting for 10% of all cancer incidences and the second leading cause of cancer-related deaths [1]. Although extensive efforts have been made to improve the diagnosis and therapy of CRC, treatment efficacy remains unsatisfactory. The primary therapeutic approaches for CRC include surgery, radiotherapy, and chemotherapy [2–4]. Chemotherapy is commonly used as a neoadjuvant therapy before surgery or as an adjuvant therapy following surgery in patients with CRC. Oxaliplatin (L-OHP) is a platinum-based drug commonly used to treat metastatic CRC. However, their effectiveness is limited by the development of drug resistance [5]. Therefore, a better understanding of the mechanisms underlying L-OHP resistance will be beneficial for successful treatment.

Several studies have suggested that transcription factors (TFs) play an important role in drug resistance. TIPE3 belongs to the tumor necrosis factor α‑induced protein 8 (TNFAIP8)‑like (TIPE) protein family that contains four members, including TNFAIP8, TIPE1, TIPE2, and TIPE3 [6]. TIPE3 acts as a transfer protein for secondary lipid messengers [7] and is associated with the progression of different types of cancers [8]. TIPE3 expression has been reported to be upregulated in several cancers, including lung, breast, and colon cancer [9–11]. However, the role of TIPE3 in drug resistance has not been identified.

The ubiquitination pathway is critical for maintaining protein homeostasis in normal physiological states and stressful environments, and its aberrant regulation has been linked to tumor cell proliferation. The catalytic activity of USP19 plays a key role in controlling tumor cell proliferation in tissues from patients with breast cancer [12]. In a mouse model study of CRC, USP19 and ERK induced the reprogramming of lipid metabolism by regulating the USP19-ME1 signaling pathway. Increased lipid metabolism is associated with ERK2 activity and colorectal carcinogenesis. Thus, the USP19-ME1 axis plays a crucial role in colorectal carcinogenesis [13]. USP24, another ubiquitinating enzyme, induces autophagy in vivo and in vitro and inhibits drug resistance in cancers acquired by taxol or gefitinib treatment [14].

Autophagy restores cellular homeostasis and prevents malignant transformation by removing damaged organelles and foreign bodies. Autophagy also promotes tumor progression by maintaining cellular stability, increasing resistance to adverse environments, and sustaining tumor growth [15]. Thus, autophagy plays a dual role in promoting and inhibiting CRC development. Patients with over- or under-expression of Beclin1 had substantially worse overall survival, suggesting that Beclin1 has a similar dual role in CRC [16]. Therefore, regulation of autophagy and Beclin1 expression could maintain tumor growth, increase resistance to chemotherapy, and promote tumor progression. In this study, we aimed to investigate the mechanism and effect of TIPE3 on CRC resistance and provide a new perspective on the potential mechanism of TIPE3 sensitivity to L-OHP.

## Results

### Expression of TIPE3 in CRC tumor tissues and cells was at high level

**Fig. 1:**
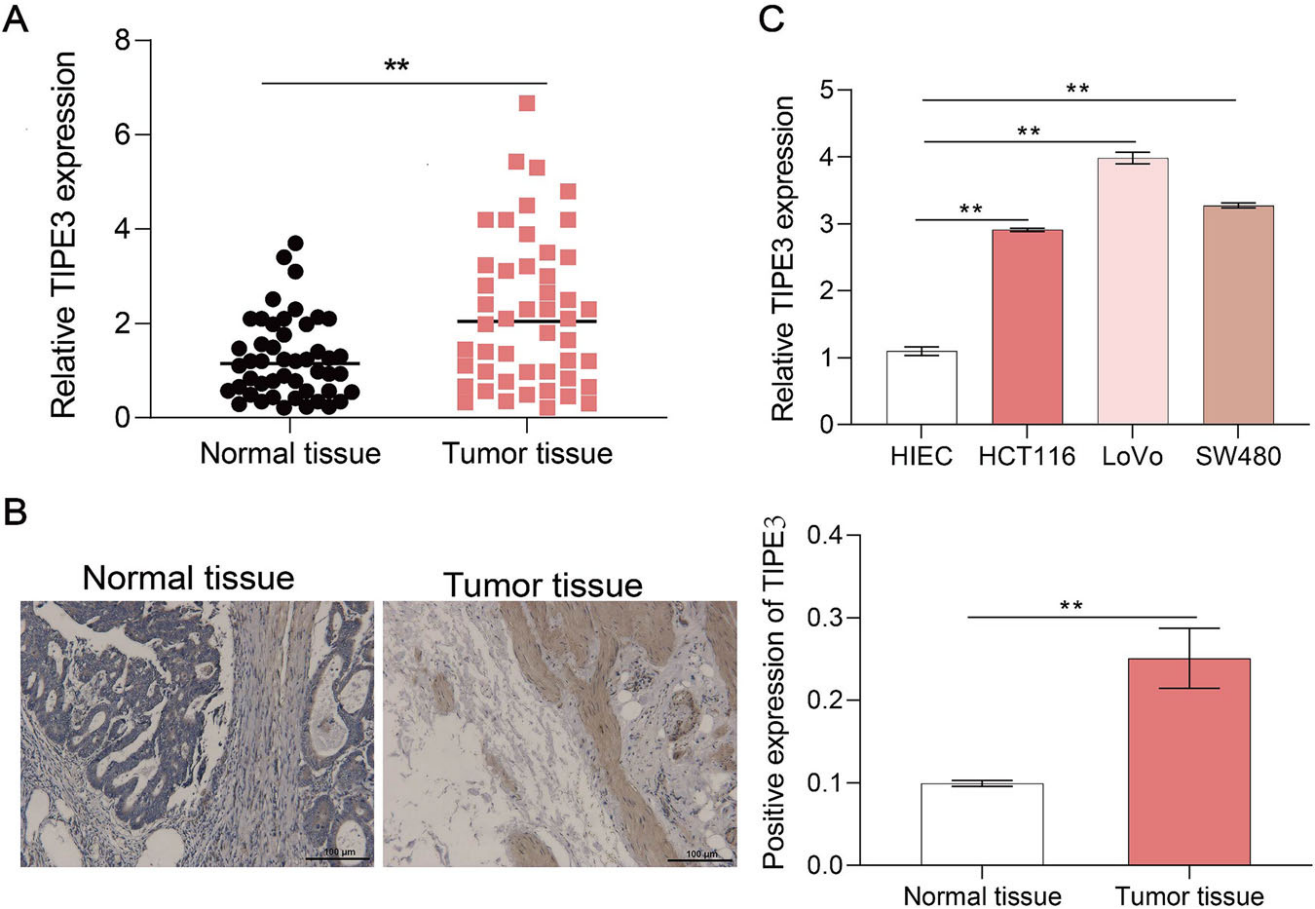
Expression of TIPE3 in CRC tumor tissues and cells. **A** RT-qPCR analysis of mRNA levels of TIPE3 (*n* = 48, *P < 0.05, **P < 0.01). **B** IHC determining the TIPE3 expression in colorectal tumors and their adjacent normal mucosa tissues. **C** qRT-PCR analysis of TIPE3 mRNA expression in HIEC, SW480, LOVO, and HCT116 cells. *P < 0.05; **P < 0.01.

A total of 48 patients were enrolled in this study. The relationship between TIPE3 expression in colorectal cancer tissues and patient clinical characteristics is shown in Supplementary Table 2. The impact on colorectal cancer patients was analyzed using Kaplan-Meier curves, as shown in Figure S1. qPCR analysis of TIPE3 expression in tumor tissues of CRC patients revealed that compared with normal tissue, tumor tissues showed markedly elevated expression of TIPE3 (Fig. 1A). In a previous study, we observed that TIPE3 was mainly localized in the cytoplasm and membranes of CC cells [17]. IHC revealed that TIPE3 expression was substantially higher in tumor tissues than in normal tissues (Fig. 1B). Additionally, TIPE3 expression was significantly higher in CRC cell lines (HCT116, LoVo, and SW480) than in normal human intestinal cells (HIECs) (Fig. 1C).

### TIPE3 promotes cell proliferation and inhibits apoptosis of CRC cells

**Fig. 2:**
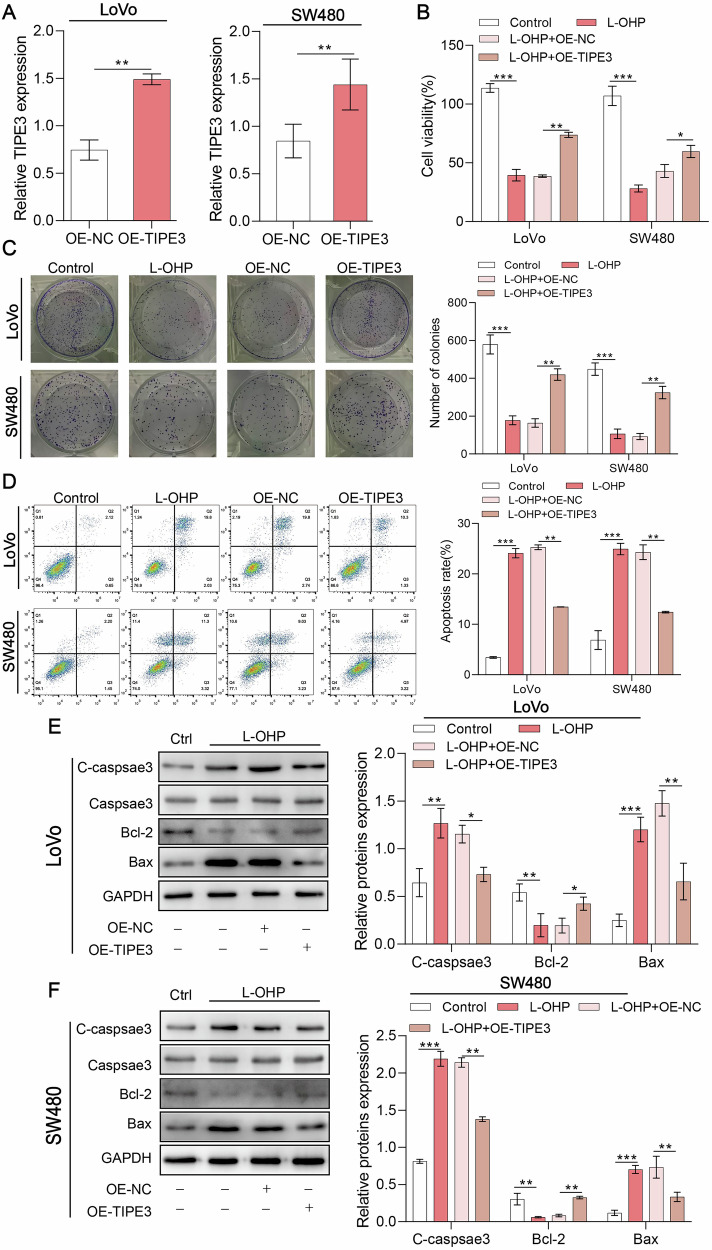
TIPE3 promotes cell proliferation and inhibits apoptosis of CRC cells. **A** qRT-PCR analysis of TIPE3 mRNA expression in SW480 and LoVo cells. **B** CCK-8 assay was carried out in TIPE3-overexpressing LoVo and SW480 cells after treatment with L-OHP. **C** Colony formation assay was performed in TIPE3-overexpressing LoVo and SW480 cells after administration with L-OHP. **D** Apoptosis was detected in TIPE3-overexpressing LoVo and SW480 cells using flow cytometry after treatment with L-OHP. **E**, **F** Western blot analysis was used to detect the expression of apoptosis-related proteins in TIPE3-overexpressing LoVo cells (**E**) and SW480 cells (**F**) after administration with L-OHP.

To verify the functional mechanism of TIPE3 in colorectal cancer, TIPE3 or control lentivirus were transfected with LoVo cells and SW480 cells. The qPCR results revealed that TIPE3 overexpression models were successfully established (Fig. 2A). The western blot results were consistent with the qPCR results (Fig. S2).

According to the results of CCK8 and colony formation assays, the OE-TIPE3 group substantially promoted cell proliferation of LoVo and SW480 cells compared to the L-OHP group (Fig. 2B, C). Additionally, TIPE3 overexpression substantially reduced the rate of apoptosis induced by L-OHP (Fig. 2D). Furthermore, the results showed that the inhibited protein levels of Bcl-2 (anti-apoptotic factor) and the elevated protein levels of Bax (pro-apoptotic factor) and cleaved caspase-3 by L-OHP were reversed in the OE-TIPE3 group in both cell lines (Fig. 2E, F). Thus, TIPE3 overexpression is beneficial for reducing apoptosis in CRC cells.

### TIPE3 increases the resistance of tumor cells to L-OHP by enhancing autophagy in CRC cells

**Fig. 3:**
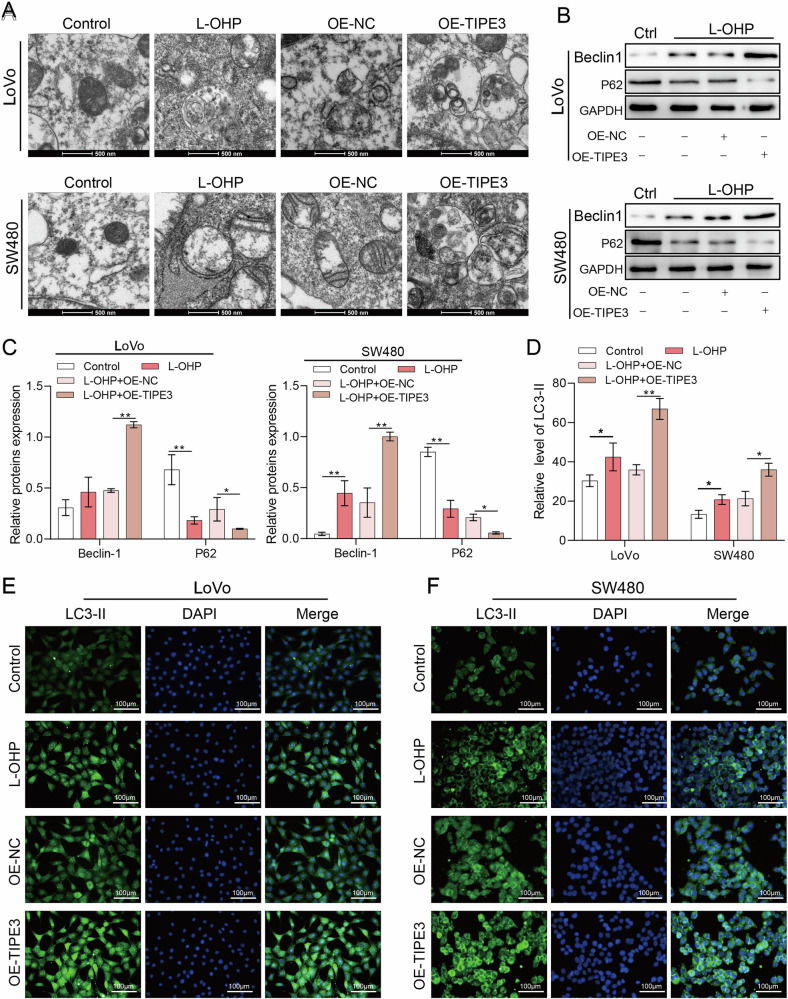
TIPE3 increases the resistance of tumor cells to L-OHP by enhancing autophagy in CRC cells. **A** Formation of autophagic vesicles in TIPE3-overexpressing LoVo and SW480 cells after administration with L-OHP was observed using transmission electron microscopy. **B**, **C** Western blot analysis was used to detect the expression of autophagy-related proteins in TIPE3-overexpressing LoVo cells and SW480 cells after administration with L-OHP. **D**–**F** Elevated levels of LC3 protein expression were detected using immunofluorescence staining in TIFE3-overexpressing LoVo cells (**E**) and SW480 cells (**F**) after treatment with L-OHP. *P < 0.05; **P < 0.01.

Next, we examined the effects of TIPE3 overexpression on autophagy in CRC cells. Transmission electron microscopy was used to observe the generation of autophagic vesicles in LoVo and SW480 cells. Compared to the L-OHP group, more autophagic vesicles were formed in the OE-TIPE3 group, with incomplete mitochondrial membranes, nuclear membranes, cytosolic membranes, and mitochondrial cristae interstitial spaces enlarged and disappeared (Fig. 3A). In addition, western blotting and immunofluorescence experiments showed that the protein expression of Beclin-1 and LC3 was significantly increased and the protein level of P62 was significantly decreased in the OE-TIPE3 group of LoVo and SW480 cells (Fig. 3B–F). These results suggest that TIPE3 overexpression enhances autophagy in CRC cells.

### TIPE3 upregulates the expression of USP19, enhancing autophagy

**Fig. 4:**
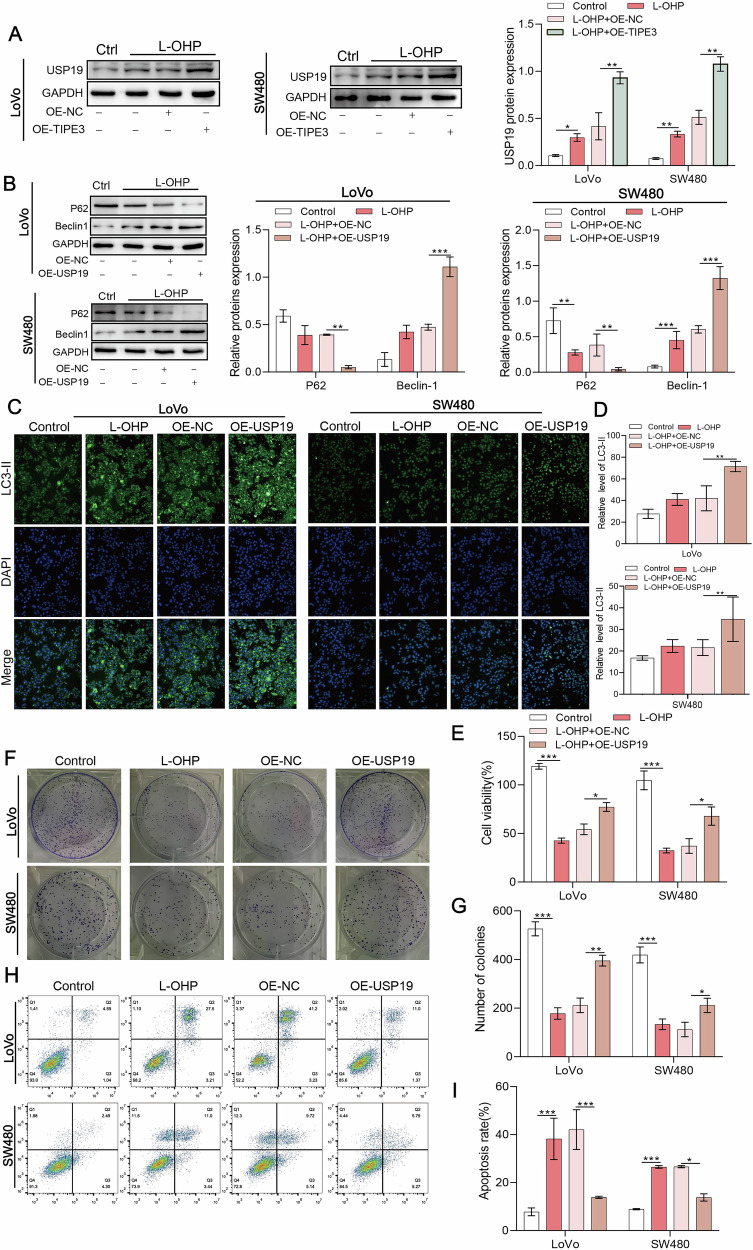
TIPE3 upregulates the expression of USP19, enhancing autophagy. **A** Western blot analysis of USP19 protein level in L-OHP treated LoVo and SW480 cells which were transduced with TIPE3 (OE-TIPE3) or control lentivirus (OE-NC). **B** Western blot analysis was used to detect the expression of autophagy-related proteins in USP19-overexpressing LoVo cells and SW480 cells after administration with L-OHP. **C**, **D** Elevated levels of LC3 protein expression were detected using immunofluorescence staining in USP19-overexpressing LoVo and SW480 cells after treatment with L-OHP. **E** CCK-8 assay was performed to determine cell growth in USP19-overexpressing LoVo and SW480 cells after administration with L-OHP. **F**, **G** Colony formation assay was performed in USP19-overexpressing LoVo and SW480 cells after administration with L-OHP. **H**, **I** Apoptosis was detected in USP19-overexpressing LoVo and SW480 cells using flow cytometry after treatment with L-OHP. *P < 0.05; **P < 0.01.

To verify the functional mechanism of TIPE3 in CRC, we investigated its effect on USP19. Western blotting results showed that TIPE3 overexpression could upregulate the expression of USP19 following L-OHP treatment in both LoVo and SW480 cell lines (Fig. 4A). Western blotting and immunofluorescence experiments of proteins related to autophagy showed that USP19 overexpression substantially reduced the protein expression of P62 in LoVo and SW480 cells while significantly elevating the protein expression of Beclin1 and LC3, as compared to the L-OHP group (Fig. 4B–D).

Moreover, the results of the CCK8 and colony formation assays (Fig. 4E–G) showed that the overexpression of USP19 recovered the damage caused by L-OHP to the cells, and the proliferative activity was enhanced. Next, overexpression of USP19 markedly reduced the apoptosis rate of cells compared to that in the L-OHP group (Fig. 4H, I). Therefore, we confirmed the effect of USP19 on drug resistance.

### Interaction of USP19 and Beclin1 induced by TIPE3 promotes cellular autophagy

**Fig. 5:**
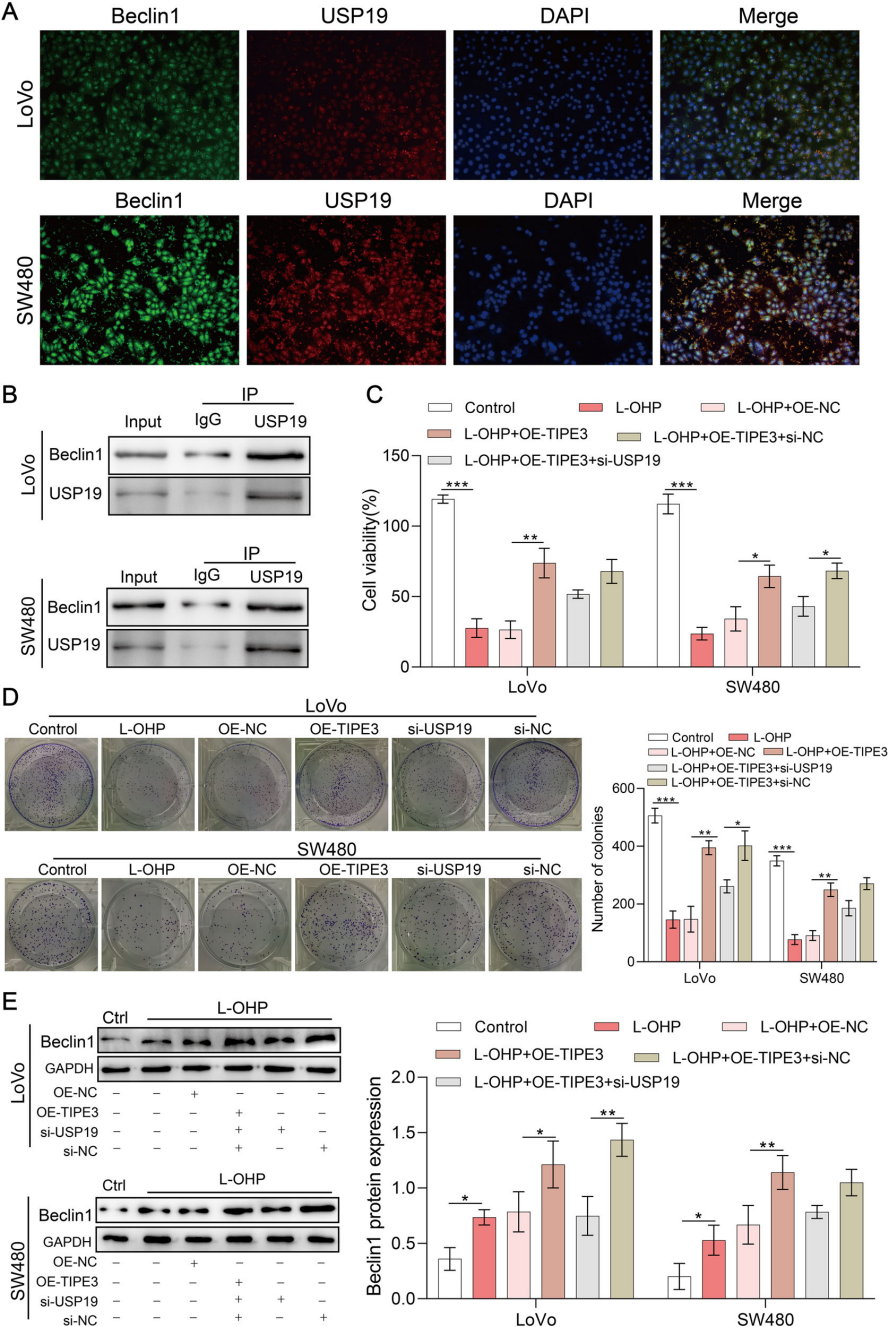
Interaction of USP19 and Beclin1 induced by TIPE3 promotes cellular autophagy. **A** The results of double immunofluorescence labeling method showed that Beclin-1 and USP19 were expressed in the nucleus, with obvious co-localization. **B** IP was used to detected the binding of USP19 and Beclin1 (key regulatory proteins of autophagy) in LoVo and SW480 cells. **C** USP19 knockdown reversed the improving effect of TIPE3-overpression LoVo and SW480 cell viability in the absence or presence of L-OHP. **D** Silencing of USP19 reversed the enhancing effect of TIPE3 overexpression on the colony formation of SW480 cells in the absence or presence of L-OHP. **E** Silencing of USP19 reversed the promoting effect of TIPE3 overexpression on Beclin1 expression in LoVo and SW480 cells after administration with L-OHP. *P < 0.05; **P < 0.01.

To investigate the regulatory role of USP19 in cellular autophagy, we examined the link between USP19 and Beclin1 (a key factor in autophagy initiation). First, USP19 and Beclin1 were localized using double immunofluorescence labeling method. Beclin-1 and USP19 were co-localized in the nucleus (Fig. 5A). Thus, an immunoprecipitation experiment was conducted to provide additional evidence for the potential interaction between USP19 and Beclin1. Beclin1 was detected among the proteins precipitated using USP19, suggesting an interaction between Beclin1 and USP19 (Fig. 5B).

Next, USP19 expression was silenced based on TIPE3 overexpression to determine the role of TIPE3 in promoting USP19 in the autophagy of cells. Figure 5C, D shows that the cell proliferation and viability enhanced by TIPE3 overexpression was inhibited by silencing USP19. Western blotting experiments showed that the elevated Beclin1 expression level after TIPE3 overexpression in LoVo and SW480 cells was decreased by USP19 silencing (Fig. 5E). In summary, TIPE3 promotes cellular autophagy by elevating the expression of USP19/Beclin1, thereby increasing drug resistance to L-OHP in CRC cells.

### TIPE3 mediates macrophagocyte M2 polarization, reinforcing drug resistance in tumor cells

**Fig. 6:**
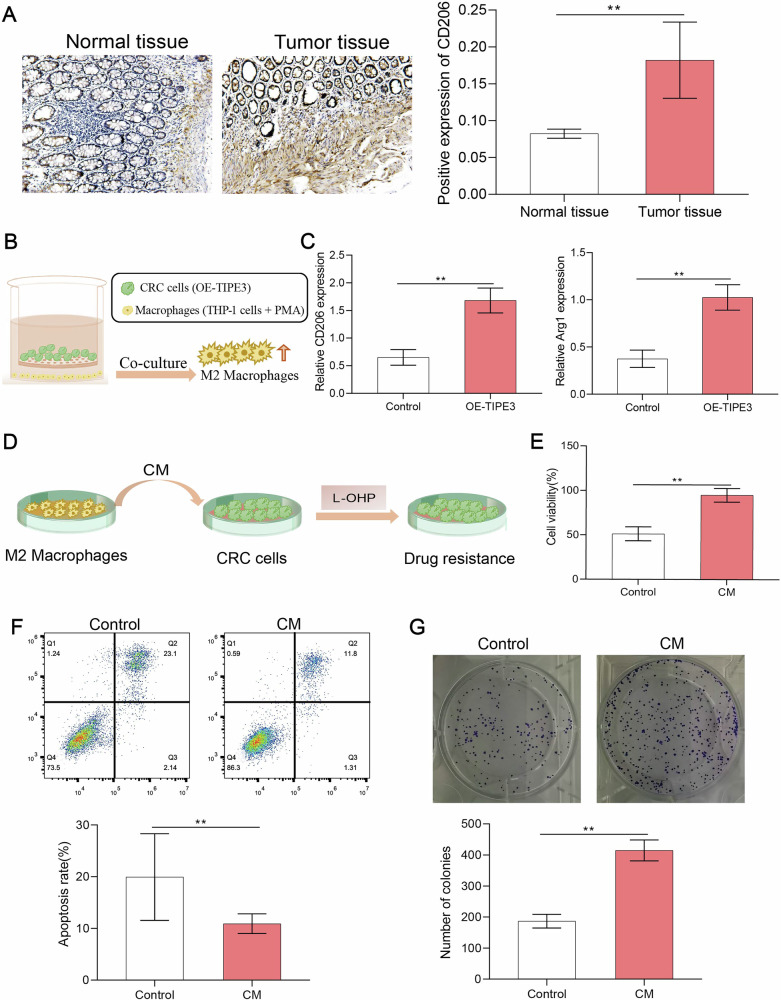
TIPE3 mediates macrophagocyte M2 polarization, reinforcing drug resistance in tumor cells. **A** IHC determining the CD206 expression in colorectal tumors and their adjacent normal mucosa tissues. **B** CRC cells that had been transfected with OE-TIPE3 were co-cultured with THP-1 cells (THP-1 cells were induced to differentiate into macrophages by PMA). The model of TIPE3 overexpression on macrophagocyte M2 polarization was created. **C** Gene expression of CD206 and Arg1 (the M2-type macrophage markers) in co-cultured cells was identified using qPCR assay. **D** THP-1 cells were induced into macrophages by PMA. Next, IL-4 was added to induce macrophages to become M2 macrophages. The medium in the dish of M2 macrophages was the conditioned medium (CM). Then, CRC cells treated with L-OHP were cultured with CM to check the drug resistance. **E** CCK-8 assay was performed to determine cell growth in CM group. **F** Apoptosis was detected in CM group using flow cytometry. **G** Colony formation assay was performed in CM group. *P < 0.05; **P < 0.01.

In the tumor microenvironment, M2-type macrophages promote tumor growth and metastasis via autophagy [18]. Therefore, we explored the effects of CRC tissues and cells on macrophage M2-type polarization. The immunohistochemical staining results showed that the expression of CD206 (an M2-type macrophage surface marker) [19] was considerably higher in the tumor tissues of patients with CRC than in normal tissues (Fig. 6A). SW480 cells that had been transfected with OE-TIPE3 were co-cultured with macrophages (THP-1 cells were induced to differentiate into macrophages by PMA) to investigate the effect of TIPE3 overexpression on macrophage M2 polarization (Fig. 6B). Gene expression of CD206 and Arg1 (M2-type macrophage markers) in co-cultured cells was determined using qPCR. The relative expression levels of CD206 and Arg1 were higher in the OE-TIPE3 group than those in the control group (Fig. 6C). This suggests that TIPE3 regulates M2 macrophage polarization.

IL-4 was added to induce macrophages (THP-1 cells + PMA) to become M2 macrophages. The medium in the dish containing M2 macrophages was conditioned medium (CM). CRC cells treated with L-OHP were cultured in CM to assess drug resistance (Fig. 6D). Cell proliferation, toxicity, and colony formation assays were performed. The results showed that cell viability was significantly enhanced in the CM group compared to that in the control group (Fig. 6E, G). In addition, the apoptosis rate in the CM group was significantly reduced (Fig. 6F). These results suggest that M2 macrophage polarization contributes to drug resistance in CRC cells.

### TIPE3 could significantly promote drug resistance to CRC in vivo

**Fig. 7:**
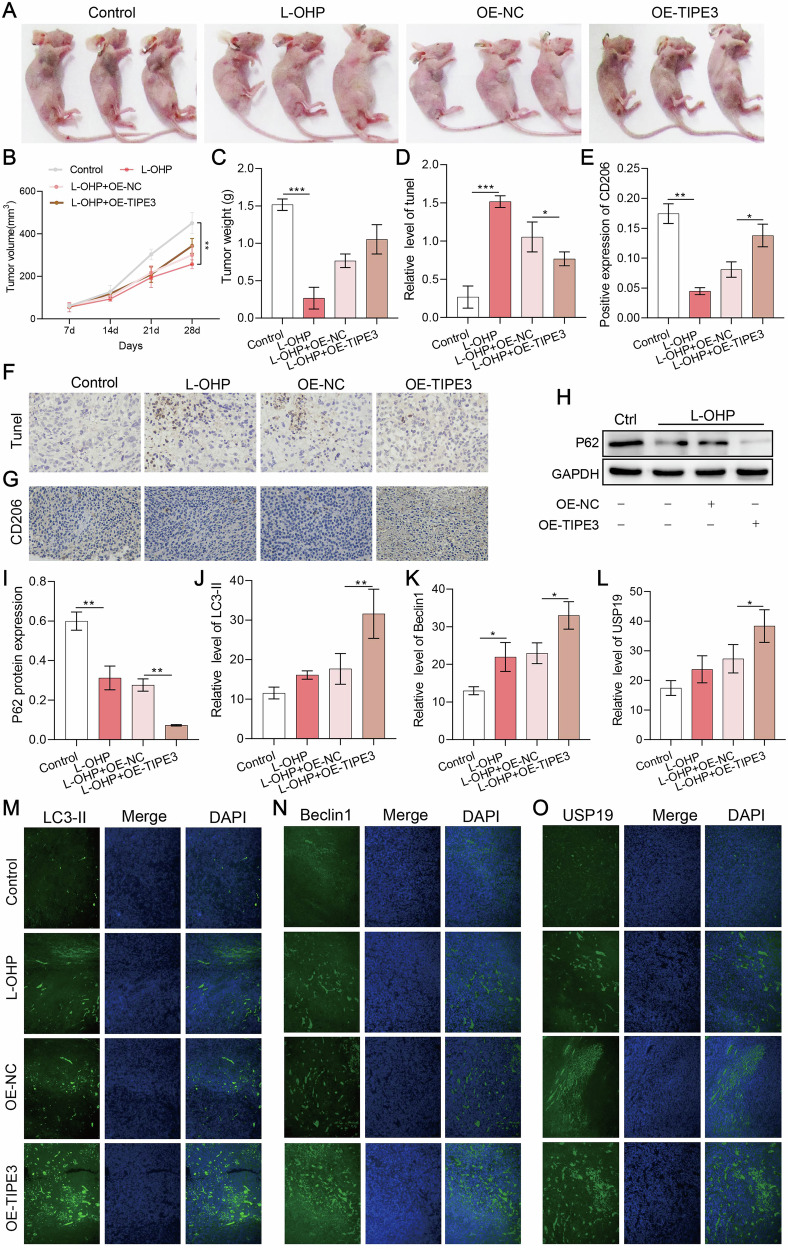
TIPE3 could significantly promote drug resistance to CRC in vivo. **A** Representative images of the nude mouse xenografts on day 28. **B** Tumor volume measured every week (*n* = 5). **C** Tumor weight on day 28 (*n* = 5). **D**, **F** Apoptosis of tumor tissues were detected using TUNEL staining (*n* = 5). **E**, **G** The expression levels of CD206 in tumor tissues were determined using IHC staining (*n* = 5). Overexpression of TIPE3 increased the expression of CD206. (**H–I**) Western blot analysis of P62 expression in TIPE3-overexpressing tumor tissues. **J**–**O** Elevated levels of LC3 (**J**, **M**), Beclin1 (**K**, **N**) and USP19 (**L**, **O**) protein expression were detected using immunofluorescence staining in TIPE3-overexpressing tumor tissues after treatment with L-OHP. *P < 0.05; **P < 0.01.

Furthermore, we validated the results obtained in the previous experiments using mice. Tumor appearance, tumor volume, and tumor weight in mice showed that TIPE3 overexpression worsened tumor growth and that the therapeutic effect of L-OHP was weaker in OE-TIPE3 group than that in L-OHP group (Fig. 7A–C). Meanwhile, TUNEL staining showed that TIPE3 overexpression significantly inhibited the apoptotic damage of tumor tissues induced by L-OHP (Fig. 7D, F), which might help tumor cells escape from the injuries caused by chemotherapeutic drugs.

However, immunohistochemical analysis showed that CD206 expression was significantly increased owing to TIPE3 overexpression compared to that in the L-OHP group (Fig. 7E, G). This suggests that TIPE3 promotes M2 macrophage polarization in mouse tumor tissues. Furthermore, western blotting (Fig. 7H, I) and immunofluorescence staining (Fig. 7J, K, M, N) showed that the protein expression of P62 was significantly reduced in the OE-TIPE3 group, whereas the protein expression of Beclin1 and LC3 was increased compared with that in the L-OHP group. In addition, consistent with the results of the experiments in LoVo and SW480 cells, TIPE3 also increased the protein expression level of USP19 in mouse tumor tissues (Fig. 7L, O).

## Discussion

L-OHP resistance is a major obstacle in the effective treatment of CRC [4]; however, the underlying molecular mechanisms remain unclear. In this study, we demonstrated that TIPE3 promotes autophagy in CRC cells and tumor-bearing mice via USP19/Beclin1 and induces macrophage M2 polarization, thus strengthening the resistance of tumor cells to L-OHP.

Several studies have demonstrated that TIPE3 is a driver gene in various cancers [9, 10, 20]. For example, TIPE3 triggers tumor progression by regulating the metabolism of phosphoinositide second messengers [21]. TIPE3 enhances the progression of lung cancer by activating Akt/mTOR, NF-κB, and STAT-3 signaling [22]. In pancreatic cancer, TIPE3 promotes tumor progression by upregulating RAC1 [23]. Our previous study showed that TIPE3 is a novel prognostic factor for CRC [11]. Our current results show a significant decrease in cell viability after L-OHP treatment. However, when TIPE3 is overexpressed, a rebound in cell viability is observed. This suggests that TIPE3 overexpression enhances cellular resistance to L-OHP. However, the function of TIPE3 in CRC remains unclear. Here, we found that TIPE3 overexpression inhibited apoptosis in CRC cells while promoting cell autophagy, alleviating drug-induced cell injury, and enhancing cellular resistance to colorectal cancer drugs. Increased TIPE3 expression is associated with regulating apoptosis, promoting inflammatory responses, and influencing the tumor microenvironment of CRC.

Autophagy plays a dual role in tumor development by inhibiting and promoting tumor growth. It has been found that USP19 promotes cervical cancer development by reducing the ubiquitination of Beclin-1. However, the effect of USP19 on CRC remains unclear, and its specific role requires further study. In addition, USP19, a typical protein in the family of ubiquitin-specific peptidases, is involved in key oncogenes or other proteins involved in tumor progression and is a potential pro- or anti-cancer factor [24, 25]. Currently, the factors upstream and downstream of USP19 that affect cancer cells remain unclear. USP19, which belongs to the category of potential pro-cancer factors, enhances the MMP2/MMP9 axis and related enzyme activities in patients with gastric cancer [25]. Therefore, the same signaling pathway may exist between TIPE3 and USP19 to achieve oncogenic effects, thereby enhancing the resistance of tumor cells to chemotherapy drugs. The expression of TIPE3 on USP19 showed an important function in the resistance to L-OHP in CRC cells. Specifically, TIPE3 upregulates the expression of USP19, reinforcing drug resistance to L-OHP in CRC cells.

In chemotherapeutic drug-treated tumor cells, enhanced autophagic activity circumvents the pressure exerted by the tumor on the cells and prevents the transformation of normal cells into tumor cells [26]. The mediating role of Beclin1 is crucial. As an important factor in the autophagy process, the elevated expression of Beclin-1 reflected that CRC cells actively initiate autophagy to remove damaged organelles and proteins [27]. Fenretinide enhances autophagic activity in MCF7/7.0.3 (human breast cancer) cell line by increasing the expression of Beclin1, thereby overcoming chemoresistance due to apoptosis and improving cancer therapy [28]. The IP results in this study showed that USP19 interacts with the Beclin1 protein to initiate autophagy in cells. Moreover, Beclin1 protein expression, which was elevated by TIPE3 overexpression, was decreased by USP19 silencing. Both phenomena indicated that the interaction between USP19 and Beclin1 induced by TIPE3 promoted cellular autophagy.

M2-type macrophages, also known as anti-inflammatory and reparative macrophages, typically play a role in tissue repair, healing, and tumor microenvironment [29]. The interaction between autophagy and M2-type macrophages is dynamically balanced and regulated by multiple signaling pathways. M2 macrophages secrete anti-inflammatory factors, inhibit immune response, and influence autophagy and apoptosis of tumor cells via paracrine mechanisms [30]. For example, CCL2 and IL-6 have definitive and potent roles in mediating both autophagy and inhibiting apoptosis within macrophages and play a key role in stimulating macrophage polarization towards the M2-type [31]. In the tumor microenvironment, macrophages are more inclined to polarize into the M2 type and promote tumor immune escape and vascular growth by secreting specific cytokines [18, 32]. In the present study, TIPE3 overexpression regulated macrophage M2-type polarization, attenuated the extent of CRC cell injury in response to L-OHP stimulation, and strengthened cell resistance to L-OHP. In vivo experiments in mice suggested that TIPE3 inhibited apoptosis of tumor cells and promoted macrophage M2-type polarization in tumor tissues by up-regulating the protein expression of USP19. Thus, TIPE3 enhances cellular resistance to colorectal cancer drugs by inhibiting the apoptosis of CRC cells, promoting autophagy, and alleviating drug-induced cellular injury.

## Conclusions

**Fig. 8:**
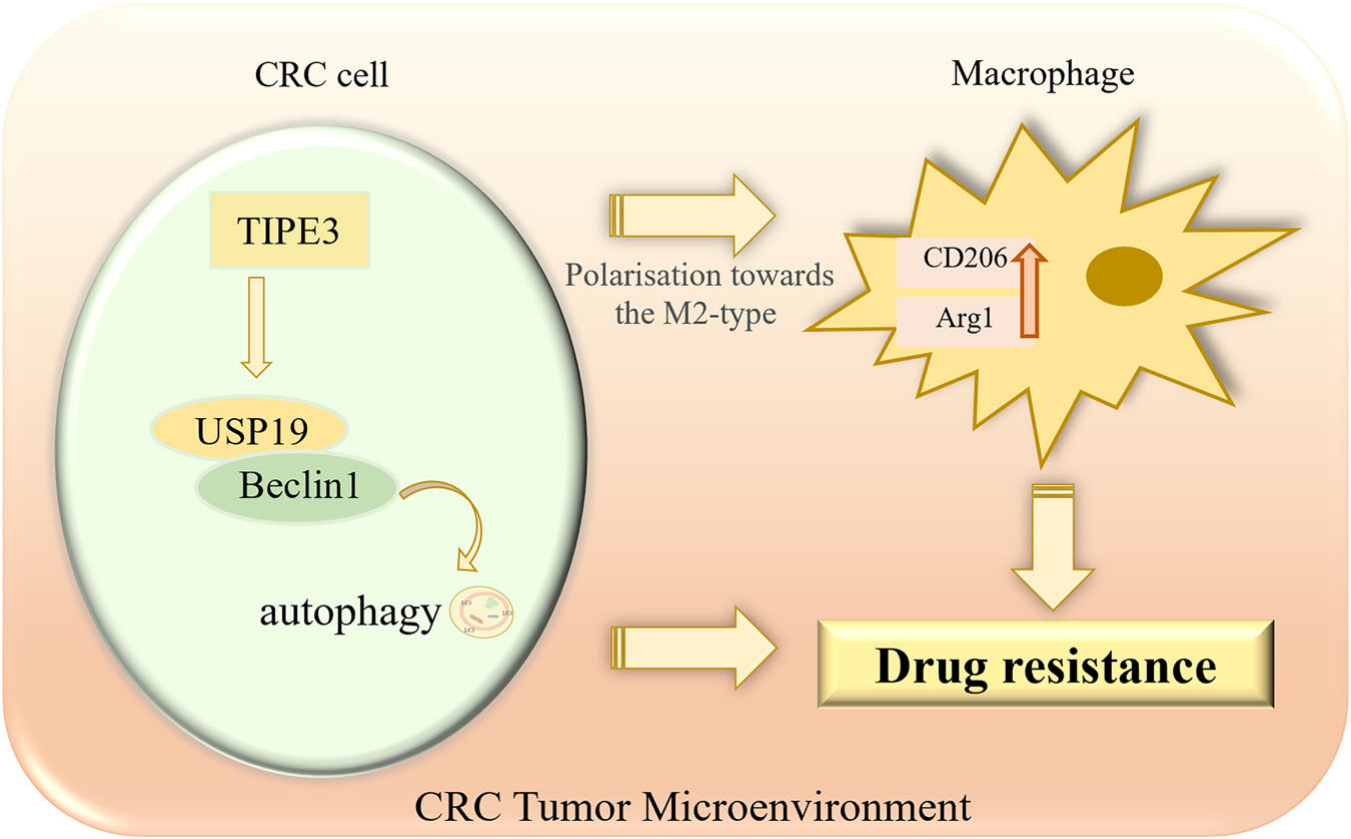
Mechanism of TIPE3 enhancing drug resistance in CRC cells. TIPE3 expression was increased in both CRC tumors and cell lines. TIPE3 upregulated USP19/Beclin1 protein expression, which successfully accelerated the autophagy process. Furthermore, TIPE3 could significantly promote drug resistance and M2-type polarization of macrophages to CRC. TIPE3 increased drug resistance by enhancing CRC cells autophagy via the USP19/Beclin1 pathway and stimulated macrophage polarization towards the M2-type.

In summary, TIPE3 regulated the expression of cell cycle- and apoptosis-related proteins through the USP19/Beclin1-pathway, thus reducing the damaging effects of L-OHP on tumor cells. Moreover, USP19 (induced by TIPE3) triggers macrophage M2 polarization in the tumor microenvironment, inhibiting the immune response and affecting autophagy and apoptosis of tumor cells (Fig. 8). TIPE3 may enhance the resistance of tumor cells to L-OHP at multiple levels, thereby affecting the efficacy of chemotherapeutic drugs. Therefore, TIPE3 may be a valuable target for the development of novel strategies to overcome L-OHP resistance.

## Materials and methods

### Cell culture and reagents

Human intestinal epithelial cell (HIEC) line and CRC cell lines (SW480, LoVo, and HCT116) were obtained from the American Type Culture Collection (ATCC; Manassas, VA, USA). HIEC and LoVo cells were cultured in Dulbecco’s Modified Eagle medium (DMEM; Gibco, Grand Island, NY, USA), and SW480 and HCT116 cells were cultured in McCoy’s 5A medium (Sigma-Aldrich, St. Louis, MO, USA) containing 10% fetal bovine serum and 1% penicillin-streptomycin and maintained at 37 °C in an incubator of 5% CO_2_. L-OHP was obtained from Sigma-Aldrich and dissolved in water to prepare a 2 mM stock solution.

### Patients and tissue samples

Primary human colorectal tumor specimens and paired non-cancerous tissues were collected from 48 patients with CRC in the Fourth Affiliated Hospital of Anhui Medical University from March 2021 to May 2022. Patients with CRC included in this study were not treated with radiotherapy or chemotherapy before surgery. This study was approved by the Medical Ethics Committee of the Fourth Affiliated Hospital of Anhui Medical University (LLSC2021010).

### Immunohistochemistry (IHC)

Colorectal tumor specimens and paired noncancerous tissues were formalin-fixed and paraffin-embedded at 4 °C overnight, and then sectioned into 10-μm-thick slices. Sections were dewaxed and dehydrated, followed by treatment with 3% hydrogen peroxide in methanol for 15 min at room temperature to quench endogenous peroxidase activity. Next, the slides were incubated with TIPE3 (diluted 1:1000; Proteintech, Rosemont, IL, USA) or CD206 (diluted 1:1000; Proteintech) antibody at 4 °C overnight prior to biotinylation with secondary antibodies at room temperature for 30 min. After staining with hematoxylin, the slides were observed under a BX-51 microscope (Olympus, Tokyo, Japan).

### siRNA transfection and lentiviral infection

To generate stable overexpression cell lines, TIPE3, USP19, or control lentivirus (Hanbio, Shanghai, China) was transduced into SW480 cells or LoVo cells and then, the cells were subsequently selected with 4 μg/mL puromycin for 10 days. To knock down TIPE3 or USP19, siRNA sequences targeting TIPE3 or USP19 (GenePharma, Shanghai, China) were transfected into SW480 or LoVo cells for 48 h. Transfection efficiency was verified using qRT-PCR and western blotting.

### CCK-8 assay

CRC cells (2 × 10^3^) were seeded in 96-well culture plates and cultured overnight prior to treatment. The culture medium was discarded, and cells were incubated in DMEM containing 10% CCK-8 (Seyotin, Guangzhou, China) for 1–4 h. The absorbance was measured at 450 nm using an epoch microplate spectrophotometer (BioTek, Winooski, VT, USA).

### Colony formation assay

Cells seeded in 6-well plates were treated with L-OHP (50 μM) for 24 h followed by trypsinization and resuspension in DMEM complete medium. Next, 500 cells/well were seeded into new 6-well plates and allowed to grow for 12–14 days. Colonies were fixed in methanol and stained with 0.5% crystal violet (Shanghai Yuanye Bio-Technology Co., Ltd., Shanghai, China) before counting under a microscope.

### Apoptosis assay

Apoptosis was stimulated with l-OHP (50 μM) in TIPE3-overexpressing, USP19-overexpressing, or USP19-silencing (si-USP19) LoVo and SW480 cells. Apoptotic cells were stained using an Annexin V-FITC/PI apoptosis detection kit (Seyotin) and detected using a flow cytometer (BD Biosciences, San Jose, CA, USA).

### Transmission electron microscopy

After stimulating with L-OHP (50 μM), autophagosomes of TIPE3-overexpressing LoVo and SW480 cells were observed using a transmission electron microscope. The cells were digested with trypsin-ethylenediaminetetraacetic acid (Beyotime Biotechnology, Shanghai, China). The cells were washed twice with ice-cold phosphate-buffered saline (PBS). Next, the cells were fixed with glutaraldehyde (2.5%, 4 °C, Merck, Darmstadt, Germany). After 12 h, the cells were fixed with 2% osmic acid solution (Merck) for 1 h and dehydrated in ethanol-acetone. Finally, the cells were sectioned and observed under an electron microscope.

### Tumor xenograft model

Animal studies, approved by the Ethics Committee of Anhui Medical University (LLSC20210488), were conducted to investigate the role of TIPE3 in tumor progression and L-OHP resistance in CRC in vivo. Twenty-four 6-week-old male nude mice were purchased from the Institute of Zoology, Chinese Academy of Sciences (Beijing, China) and randomly divided into four groups. SW480 cells transfected with lentiviral TIPE3 plasmids or empty vectors were subcutaneously injected into the flanks of the mice. Seven days later, 5 mg L-OHP/kg body weight or normal saline was subcutaneously administered every 3 days. The tumor volume was measured every 3 days. On day 30, all mice were sacrificed, and tumor specimens were harvested, weighed, and subjected to the TUNEL assay and IHC.

### TUNEL assay

Apoptosis was analyzed using the TUNEL assay with an In Situ Cell Death Detection Kit (Roche Applied Science, Penzberg, Germany). Tumor specimens from mice were formalin-fixed and paraffin-embedded at 4 °C overnight, and then sectioned to 4-μm-thick slices. The sections were then deparaffinized and dehydrated. After washing with PBS, the cells were permeabilized in 0.2% Triton X-100 for 20 min. Then the slides were incubated with TUNEL reaction mixture in a humidified chamber for 60 min at 37 °C according to the manufacturer’s instructions. The slides were counterstained with hematoxylin, and apoptotic cells were counted under a light microscope.

### Quantitative real‑time PCR assay (qRT-PCR)

Total RNA was extracted from CRC cells using the TRIzol reagent (Invitrogen, Carlsbad, CA, USA). Two micrograms of RNA from each sample were used for cDNA synthesis using a Reverse Transcription Kit (Seyotin), according to the manufacturer’s instructions. qPCR was performed in 20 µL volume using qPCR mix (Seyotin) on a StepOnePlus Real-Time PCR System (Applied Biosystems, Foster City, CA, USA). The mRNA level of β-actin was served as a control. The primers used are listed in Supplementary Table 1.

### Western blot analysis

Tissue samples and whole cells were lysed in RIPA lysis buffer (Seyotin) supplemented with a protease inhibitor cocktail. Protein samples were prepared following ultrasonication and centrifugation at 4 °C. Twenty micrograms of protein from each sample were separated using SDS-PAGE and transferred to PVDF membranes (Millipore; Billerica, MA, USA). After blocking, the membranes were probed with the following monoclonal antibodies (Abcam, Cambridge, UK): Cleaved-caspase 3(1:1000), caspase 3 (1:1000), Bcl-2 (1:1000), Bax (1:1000), Beclin1 (1:1000), P62 (1:1000), USP19 (1:1000) and GDPDH (1:10000) overnight at 4 °C, followed by incubation with secondary antibodies (1:2000, Abcam) for 2 h at room temperature. The membranes were washed thrice with TBST and detected using an ECL detection kit (Seyotin).

### Immunofluorescence staining

Immunofluorescence staining was performed to probe the interaction between USP19 and Beclin1 promoter. SW480 and LoVo cells were seeded at a density of 1 × 10^7^ cells/well and cultured overnight. The cells were then immobilized with a potassium permanganate solution (4%; Merck) and permeabilized with a PBS solution of 0.1% Triton X-100. After blocking, the cells were bathed in LC3 (1:1000), Beclin1 (1:1000), and USP19 (1:1000) antibody solution (4 °C, overnight; Abcam, Cambridge, UK), followed by incubation with secondary antibodies with fluorescent markers (1:2000; Abcam) for 1 h at room temperature and avoiding light. After staining the nuclei using DAPI (Beyotime Biotechnology), the slides were encapsulated with a fade-resistant sealer. Fluorescent signals were observed and recorded using a fluorescence microscope to localize and quantify the distribution of proteins within the cells.

### Differentiation of THP-1 cells into macrophages

THP-1 cells (ATCC, Manassas, VA, USA) were seeded in cell culture dishes at an appropriate density (5 × 10^5^ cells/ mL) and cultured at 37 °C with 5% CO_2_. Next, phorbol 12-myristate 13-acetate (PMA, 100 nM; Shanghai Yuanye Bio-Technology Co., Ltd., Shanghai, China) was added and the cells were cultured for 48–72 h. During this process, the THP-1 cells gradually stopped proliferating and began to adhere. The surface morphology then changed from a rounded shape to an irregular shape, showing the typical characteristics of macrophages. After removing PMA, IL-4 was added to the medium (15 ng/mL, 72 h). At this point, the macrophages were stimulated to polarize towards the M2 type.

### Statistical analysis

Data are shown as mean ± standard deviation. Student’s *t* test was used to determine statistical significance between two groups. When more than two groups were compared, a one-way analysis of variance was used, followed by the Bonferroni post-hoc test using GraphPad Prism 6.0 (GraphPad Software, San Diego, CA, USA). Statistical significance was set at a value of *P* < 0.05.

## Supplementary information

Supplementary Material

Full length western blots

## Data Availability

The data are available from the corresponding author upon reasonable request.
