# Supplementary material for: TIPE3 promotes drug resistance in colorectal cancer by enhancing autophagy via the USP19/Beclin1 pathway
Source: Cell Death Discov. 2025 Apr 25;11:202. doi: 10.1038/s41420-025-02477-x (PMC12032075; doi:10.1038/s41420-025-02477-x)
Supplement: Supplementary file 1 — Supplementary Material [file 41420_2025_2477_MOESM1_ESM.docx]

**TIPE3** **promotes** **drug resistance in colorectal cancer by enhancing autophagy via the USP19/Beclin1 pathway**

Chun Chen^1,2*^, Longyang Jin^2*^, Hong Wan^4^, Hu Liu^4^, Shuping Zhang^5^, Gang Shen^6&^, Jiao Gong^6&^, Yong Zhu^3,4&^

**Supplementary results**

**Supplementary Figure S1. Kaplan Meir curve for patients with colorectal cancer.**

**
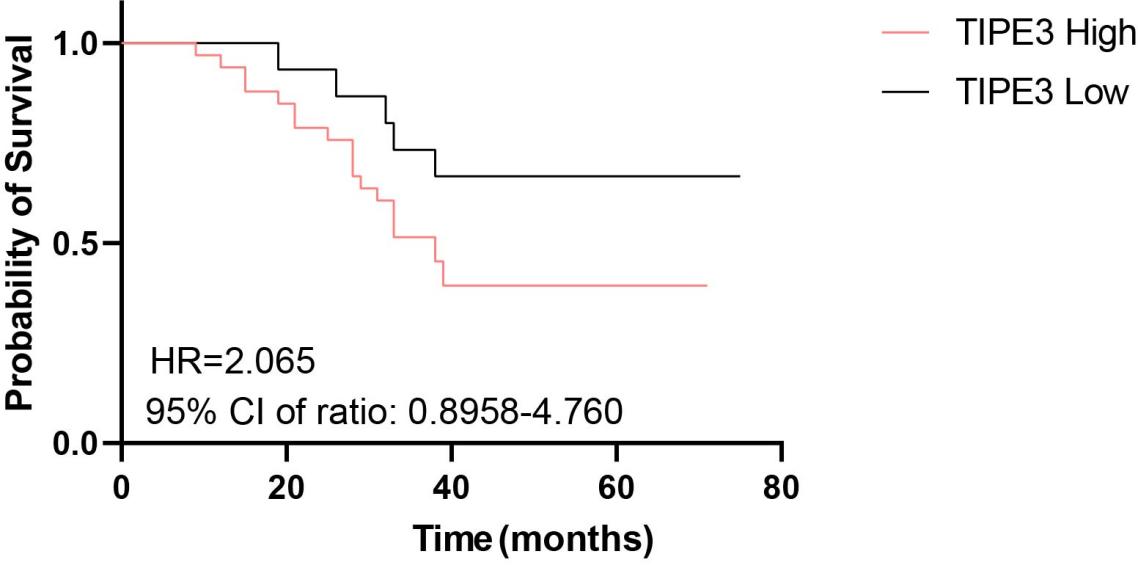
**

**Supplementary Figure S2. TIPE3 promotes cell proliferation and inhibits apoptosis of CRC cells. (A)** Western blot analysis of TIPE3 protein expression in SW480 and LoVo cells.


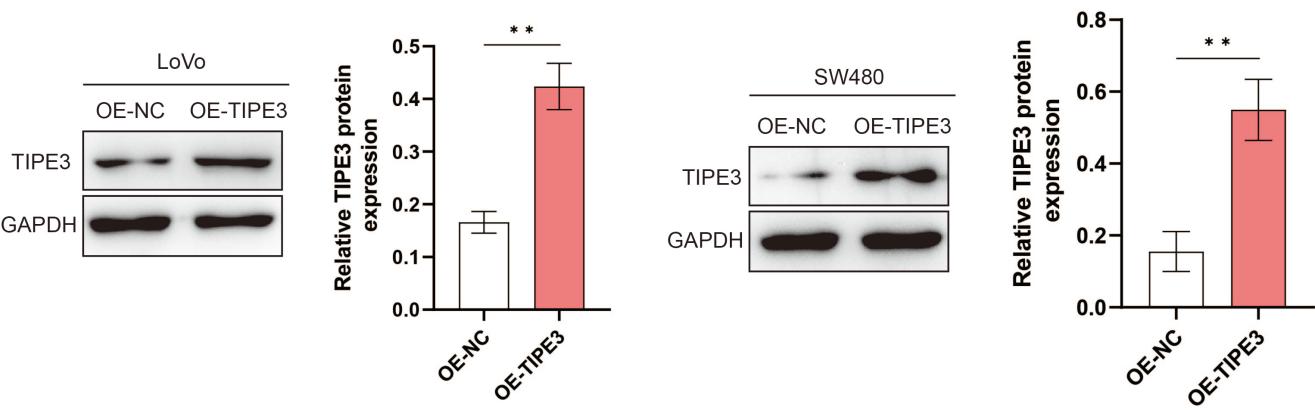


**Supplementary Table 1. Primer sequence of qPCR.**

| Primer | Primer sequence | | Length of product/bp |
| --- | --- | --- | --- |
|  | Forward sequence (5’-3’) | Reverse sequence (5’-3’) |  |
| TIPE3 | CCGCAGCATGGATTCGGATT | GTTGGCCACAGTTTTGCTGG | 145 |
| CD206 | GCAAAAAGGGCAACACCACT | TTCCTTCCTGCAGGTGGTCA | 164 |
| Arg1 | GAGTCATCTGGGTGGATGCT | CAGGGAGTCACCCAGGAGAA | 148 |
| β-actin | CAGGGAGTCACCCAGGAGAA | CAGGGAGTCACCCAGGAGAA | 136 |

**Supplementary Table 2 Association between TIPE3 expression in CRC tissues and clinical features of patients**

| Clinicopathological | case | TIPE3 expression | |
| --- | --- | --- | --- |
|  |  | High | Low |
| Ages |  |  |  |
| 60 or Less | 22 | 14 | 8 |
| >60 | 26 | 19 | 7 |
| Gender |  |  |  |
| Males | 30 | 20 | 10 |
| Females | 18 | 13 | 5 |
| Differentiation |  |  |  |
| Moderate | 37 | 24 | 13 |
| Poor | 11 | 9 | 2 |
| metastasis |  |  |  |
| No | 31 | 22 | 9 |
| N1 or more | 17 | 11 | 6 |
