# Supplementary material for: TIPE3 promotes drug resistance in colorectal cancer by enhancing autophagy via the USP19/Beclin1 pathway
Source: Cell Death Discov. 2025 Apr 25;11:202. doi: 10.1038/s41420-025-02477-x (PMC12032075; doi:10.1038/s41420-025-02477-x)
Supplement: Supplementary file 2 — Full length western blots [file 41420_2025_2477_MOESM2_ESM.doc]

| Figure 2 E | | Figure 2 F | |
| --- | --- | --- | --- |
| C-caspase3 | 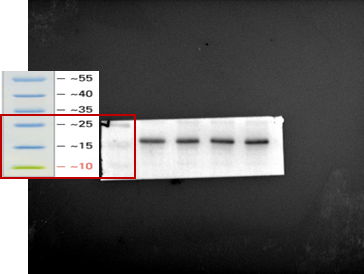 | C-caspase3 | 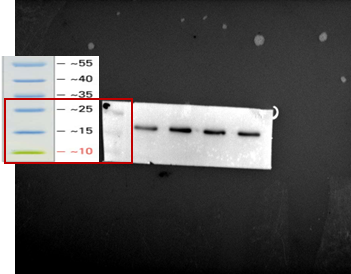 |
| Caspase3 | 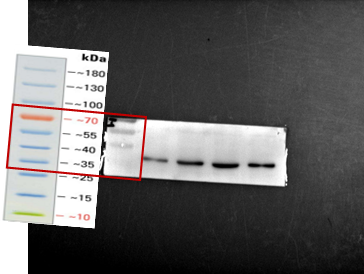 | Caspase3 | 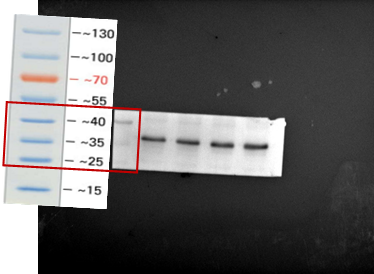 |
| Bcl-2 | 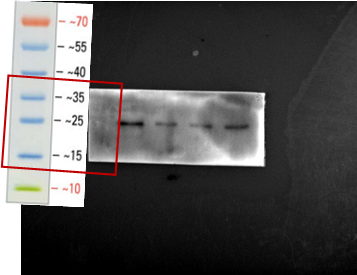 | Bcl-2 | 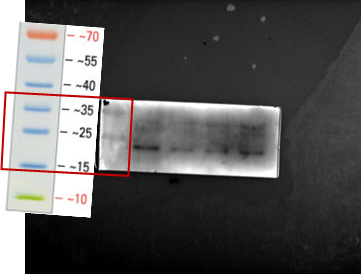 |
| Bax | 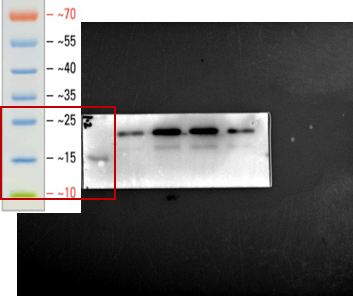 | Bax | 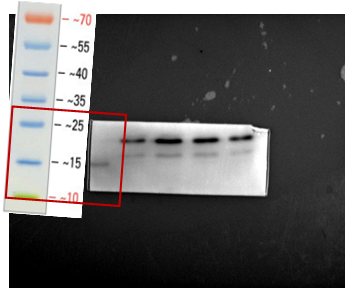 |
| GADPH | 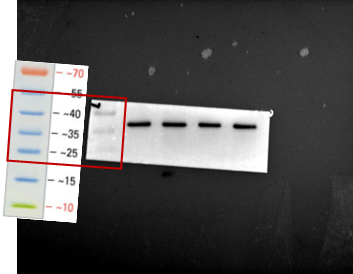 | GADPH | 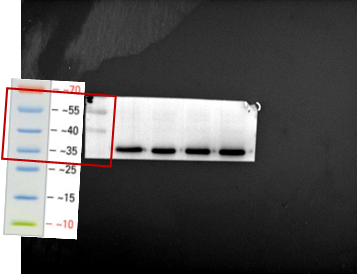 |

| Figure 3B | | | |
| --- | --- | --- | --- |
| LoVo cell | | SW 480 cell | |
| Beclin1 | 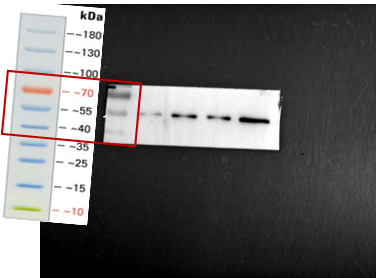 | Beclin1 | 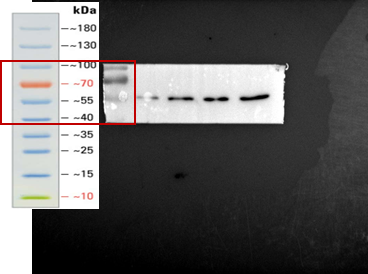 |
| P62 | 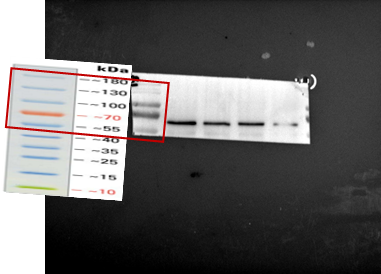 | P62 | 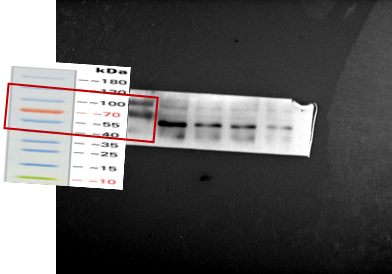 |
| GADPH | 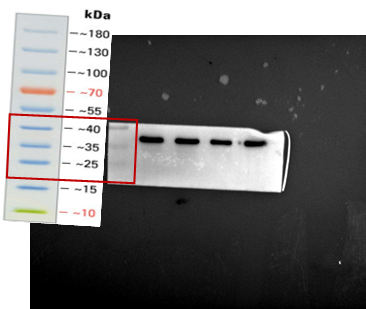 | GADPH | 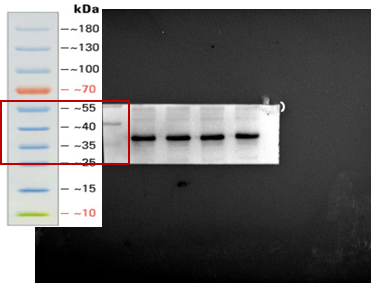 |

| Figure 4A | | | |
| --- | --- | --- | --- |
| LoVo cell | | SW 480 cell | |
| USP19 | 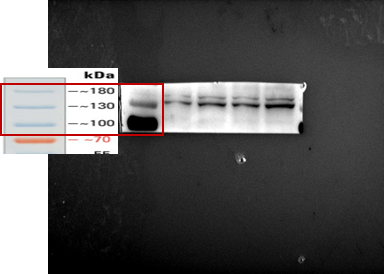 | USP19 | 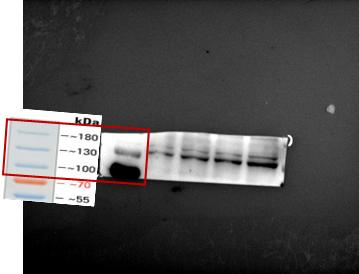 |
| GADPH | 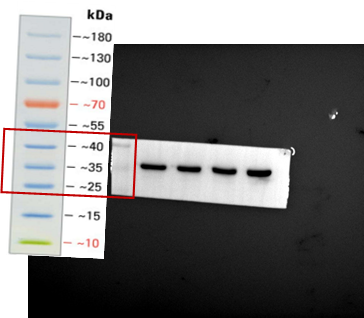 | GADPH | 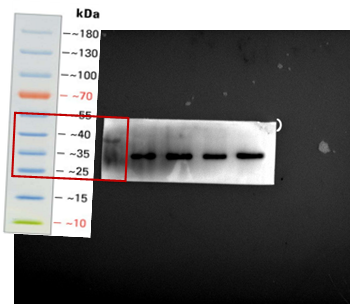 |

| Figure 4B | | | |
| --- | --- | --- | --- |
| LoVo cell | | SW 480 cell | |
| Beclin1 | 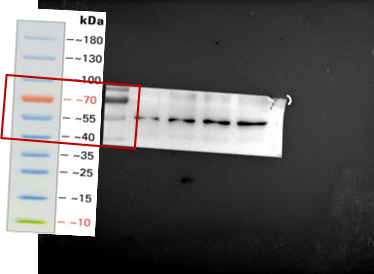 | Beclin1 | 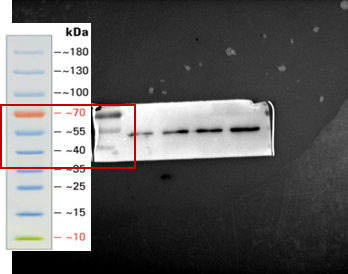 |
| P62 | 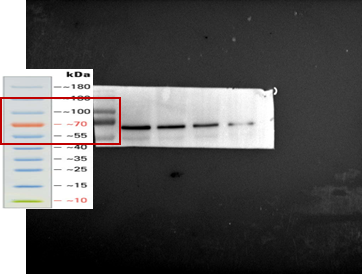 | P62 | 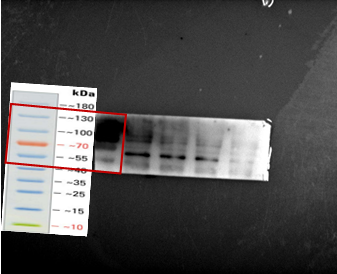 |
| GADPH | 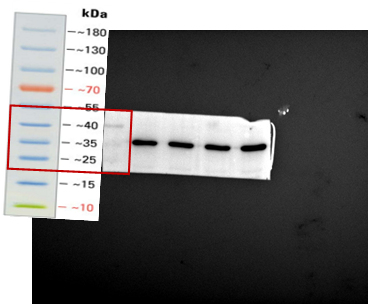 | GADPH | 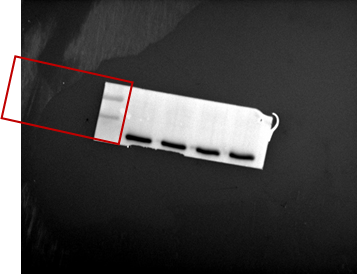 |

| Figure 5A | | | |
| --- | --- | --- | --- |
| LoVo cell | | SW 480 cell | |
| USP19 | 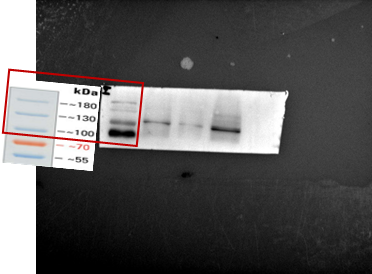 | USP19 | 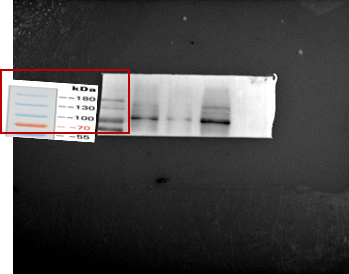 |
| Beclin1 | 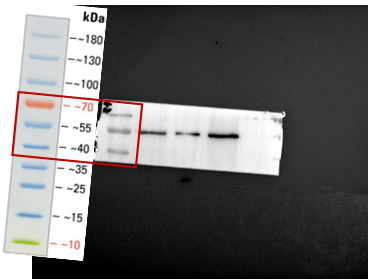 | Beclin1 | 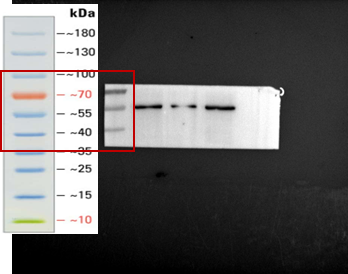 |

| Figure 5E | | | |
| --- | --- | --- | --- |
| LoVo cell | | SW 480 cell | |
| Beclin1 | 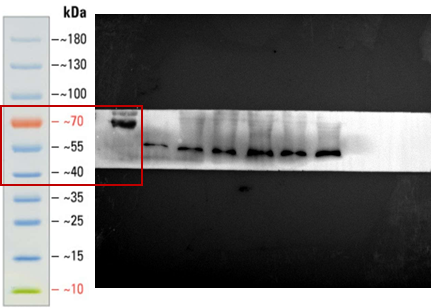 | Beclin1 | 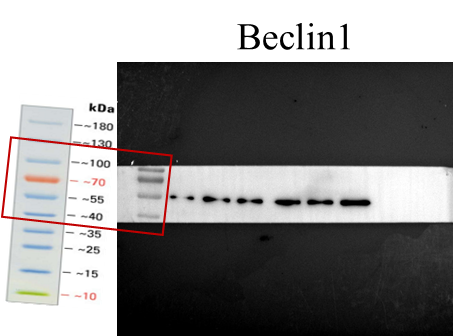 |
| GADPH | 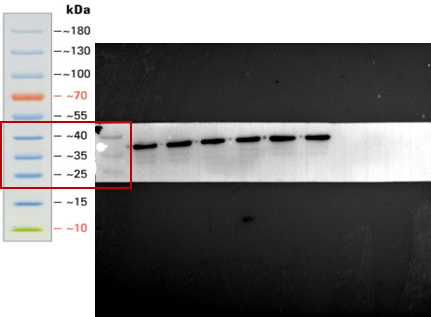 | GADPH | 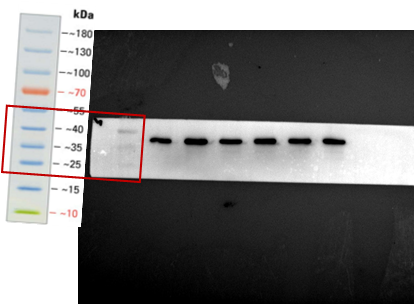 |

| Figure 7H | | | |
| --- | --- | --- | --- |
| P62 | 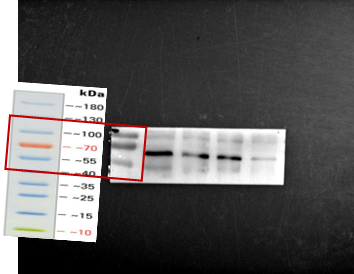 | GADPH | 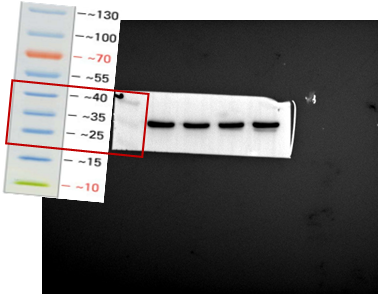 |
